# From Intracoronary Physiology to Endotype-Based Treatment: Quality of Life Improvement for INOCA Patients

**DOI:** 10.3390/jcm14207192

**Published:** 2025-10-12

**Authors:** Barbara Vitola, Laima Caunite, Karlis Trusinskis, Iveta Mintale, Andrejs Erglis

**Affiliations:** 1 Latvian Centre of Cardiology, Pauls Stradins Clinical University Hospital, LV-1002 Riga, Latvia; karlis.trusinskis@gmail.com (K.T.); iveta.mintale@stradini.lv (I.M.); a.a.erglis@stradini.lv (A.E.); 2 Department of Doctoral Studies, Riga Stradins University, LV-1007 Riga, Latvia; 3 Department of Cardiology, Leiden University Medical Center, Albinusdreef 2, 2333 ZA Leiden, The Netherlands; l.caunite@lumc.nl; 4 Faculty of Medicine and Life Sciences, University of Latvia, LV-1586 Riga, Latvia

**Keywords:** INOCA, microvascular angina, vasospastic angina, quality of life, intracoronary physiology, coronary flow reserve, acetylcholine provocation test

## Abstract

**Background/Objectives**: Ischemia with non-obstructive coronary arteries (INOCA) remains an underdiagnosed and undertreated condition due to the extensive diagnostic testing required and heterogeneous pathophysiology of different endotypes, each of which require tailored treatment. This study aimed to explore the effect of intracoronary physiology testing-based endotype-specific medical therapy on quality of life in patients with INOCA. **Methods**: Intracoronary physiology testing was performed in patients presenting with cardiac symptoms, evidence of significant ischemia on non-invasive testing, and non-obstructive epicardial coronary arteries. Microvascular angina (MVA) was defined as coronary flow reserve ≤ 2.5 and an index of microvascular resistance ≥ 25. Vasospastic angina (VSA) was defined as a >90% vasoconstriction of an epicardial artery during acetylcholine provocation test in the presence of ischemic electrocardiogram changes and chest pain. Quality of life was evaluated using the Seattle Angina Questionnaire 7 (SAQ-7) before the start of new treatment and at the three months follow-up. **Results**: The total study population consisted of 35 patients (80% women), of whom MVA was observed in 19 (54.3%), VSA in 9 (25.7%), and the combination of MVA and VSA in 3 (8.6%) cases. Four patients (11.4%) had no pathology on intracoronary physiology testing detected. High rates of dyslipidemia (100%), arterial hypertension (85.7%), diabetes (17.1%), and depression and anxiety (34.3%) were documented. In the isolated MVA and VSA groups, adjustment of medical therapy resulted in an improvement in the SAQ-7 summary score at 3 months (*p* < 0.001 and *p* = 0.007, respectively). There was no change of SAQ-7 summary score in the mixed endotype group (*p* = 0.11). **Conclusions**: Adjustment of medical therapy according to intracoronary physiology testing-based phenotype resulted in improved quality of life as assessed by the SAQ-7. Our findings highlight the importance of invasive testing in patients with clinically suspected INOCA.

## 1. Introduction

Myocardial ischemia is the primary underlying mechanism of adverse events associated with coronary artery disease (CAD), including mortality, and a major contributor to reduced quality of life (QoL) [1]. CAD has traditionally been diagnosed based on the presence of obstructive lesions; however, approximately 40% of stable angina patients undergoing elective invasive coronary angiography (ICA) have non-obstructive coronary arteries [2]. While decades of advancements have focused on identifying and treating atherosclerotic coronary plaque-induced narrowing, other factors may also contribute to myocardial ischemia, regardless of significant stenosis. A key factor among these is the dynamic function of the coronary microcirculation [3,4,5,6]. Given that research and understanding of this patient’s group has grown considerably, the Coronary Vasomotor Disorders International Study Group (COVADIS) criteria and European Society of Cardiology (ESC) chronic coronary syndrome guidelines now recommend that ischemia with non-obstructive CAD (INOCA) be considered a likely diagnosis [7,8].

INOCA is associated with increased risk of major adverse cardiac events, such as frequent re-hospitalizations due to chest pain, stroke and heart failure with preserved ejection fraction [9,10], and mortality rates comparable to those of obstructive CAD [11], especially in patients who remain symptomatic [12].

INOCA can be caused by heterogenous mechanisms, including both endothelial- and non-endothelial-dependent coronary microvascular dysfunction (CMD) and epicardial or microvascular coronary vasospasm [13]. Accordingly, INOCA is classified as microvascular angina (MVA), vasospastic angina (VSA), or a combination of both [6].

Extensive intracoronary physiology testing of INOCA is crucial for an accurate diagnosis [6,8]. However, often these tests are not routinely performed due to their complex and time-consuming protocols, which require specially trained personnel and advanced cardiac catheterization laboratory equipment, and also limited awareness about the benefits of a clear diagnosis including the INOCA endotype [14,15].

As the guideline-directed medical treatment differs between MVA and VSA endotypes [8,14], lack of definite classification leads to suboptimal treatment and outcomes [16].

Our aim was to investigate the prevalence of various INOCA endotypes, characterize key clinical features of the patient cohort, identify modifications in medical therapy following definitive diagnosis, and assess changes in quality of life after therapeutic adjustments.

## 2. Materials and Methods

### 2.1. Study Design and Population

This was a single-center observational prospective cohort study. The study enrolled all patients referred to an interventional diagnostic procedure (IDP) in Pauls Stradins Clinical University Hospital, Latvian Centre of Cardiology from March 2023 until December 2024. Referral criteria were (1) symptoms of myocardial ischemia: effort and/or rest angina or angina equivalents (i.e., shortness of breath); (2) objective evidence of myocardial ischemia: positive exercise stress ECG or true myocardial perfusion defect in cardiac single photon emission tomography, which was defined as area of ischemia ≥ 10% of the left ventricle myocardium; and (3) confirmed absence of obstructive CAD by coronary computed angiography or ICA. Clinical data were collected at the time of inclusion and available in all patients. Blood tests were drawn and analyzed as per hospital standard operating protocol prior to the planned IDP. Medical therapy followed the latest ESC guidelines for chronic coronary syndromes [8]. Patients with MVA treatment recommendations included angiotensin-converting enzyme inhibitors, vasodilatory beta blockers (BBs), calcium channel blockers (CCBs), statins, and antianginal medication like trimetazidine or ranolazine. For the VSA patient group, CCBs, statins, and long-acting nitrates were recommended.

This study was approved by the ethics committee of Riga Stradins University (No. 2-PEK-4/472/2022) and conducted in compliance with the Declaration of Helsinki. Each patient gave written informed consent.

### 2.2. The Interventional Diagnostic Procedure

The radial approach with a 6-F guide catheter was used in 32 (91.4%) patients. To avoid confounding, use of vasodilatory drugs was avoided. The left anterior descending coronary artery was chosen as a target artery for testing.

The IDP was performed as a two-step approach. First, the microvascular function was conducted using The Pressure X Guidewire (Abbot, Abbott Park, North Chicago, IL, USA) coronary guidewire and Coroventis Coroflow (Coroventis Research AB, Uppsala, Sweden) system by inducing maximal hyperemia with 140 µg/kg/min of intracoronary adenosine and measuring the fractional flow reserve (FFR), coronary flow reserve (CFR), and index of microvascular resistance (IMR).

Second, the acetylcholine provocation test was performed to assess coronary vasospasm. Acetylcholine solutions in doses of 2 µg, 20 µg, and 100 µg up to the maximal concentration of 200 µg were slowly infused directly in the coronary artery. Coronary angiogram was performed after every dose. During the test the 12-lead ECG was continuously monitored. At the end of the intracoronary physiology testing, 300 µg of glyceryl trinitrate was administered.

### 2.3. Diagnostic Criteria for INOCA Endotypes

Following the IDP, patients were classified into four groups: MVA, VSA, combined MVA and VSA, and non-cardiac chest pain. MVA was confirmed when CFR ≤ 2.5, IMR ≥ 25, and FFR > 0.8. VSA was confirmed when a significant reduction in the coronary artery lumen diameter (>90%) was observed, accompanied by angina symptoms or ischemic ECG changes. If no vasospasm was observed but patient developed angina symptoms and ischemic ECG changes, the test was considered positive for microvascular vasospasm. The patients with defined microvascular vasospasm were included in the MVA group. If a patient tested positive for both endotypes, they were assigned to the combined endotype group. Chest pain was considered non-cardiac if all performed tests were negative.

### 2.4. Outcomes

The primary outcome was changes in the Seattle Angina Questionnaire (SAQ-7). It includes questions about physical limitations, angina stability, angina frequency, and QoL [17]. Scores range from 0–100, with higher values indicating fewer symptoms and better health status. All patients were invited to complete the questionnaire at baseline and at the three-month follow-up, which was conducted via phone interview.

### 2.5. Statistical Analysis

Due to the very small subgroup size, all continuous data were treated as nonparametric and are presented as median and interquartile range if number of patients ≥ 5 or median and range if number of patients < 5, as appropriate. Categorical data are presented as frequencies and percentages. Continuous variables between the groups were compared with Kruskal–Wallis tests. Categorical variables were compared with chi-square tests or the Fisher exact test as appropriate. Changes in SAQ score over time within the groups were compared using the Wilcoxon signed-rank test for continuous variables and the McNemar test for categorical variables. Bonferroni correction was consistently applied for all multiple subgroup comparisons in the post hoc analysis. Statistical analysis was performed using SPSS version 29.0 (IBM, Armonk, New York, NY, USA) and R version 4.5.0 (R Foundation for Statistical Computing, Vienna, Austria). All statistical tests were two-sided, and a *p* value < 0.05 was considered statistically significant.

## 3. Results

### 3.1. Clinical Characteristics, Symptoms and Risk Factors

Total study population was 35 patients (mean age 61.0 years, 28 (80.0%) women). Baseline characteristics are shown in Table 1. The most common complaints were chest pain/discomfort/chest pressure (n = 32; 91.4%), shortness of breath (n = 28; 80.0%), fatigue/exhaustion (n = 23; 65.7%), headache (n = 14; 40.0%), and excessive sweating (n = 10; 40.0%). More than 25% suffered from anxiety (n = 9; 25.7%). There were no differences in symptoms between INOCA groups, except that the rest angina was more common in VSA patients (88.9% vs. 10.5% in MVA group; *p* < 0.001).

Arterial hypertension was present in 30 (85.7%) patients, diabetes mellitus in 6, (17.1%) and 5 (14.3%) were current or ex-smokers. Twelve (34.3%) patients had a known history of depression or anxiety. All 35 patients had dyslipidemia, 22 (62.9%) of whom received lipid-lowering therapy before inclusion in the study. All but one of the smokers presented with a VSA endotype (*p* = 0.014). Laboratory data did not differ between groups.

### 3.2. Referral Pattern and Previous Examination Tool Analysis

Most (n = 26; 83.9%,) of the study patients had experienced INOCA symptoms for more than one year and four (11.4%) had been symptomatic for more than five years. The majority (n = 31; 88.6%) of patients had visited at least two cardiologists, 17 had been admitted to the emergency department, and five (14.2%) patients had presented to the emergency department more than two times with these complaints.

Referral patterns and previous investigation before invasive testing are shown in Table 2. All study patients had undergone previous ECG, exercise ECG, and echocardiography. Because of limited availability, the cardiac single-photon emission tomography or positron emission tomography was performed only in four (11.5%) patients. In contrast, the majority of patients (n = 30; 85.7%) had been previously referred to ICA and 17 (48.6%) had undergone ICA twice prior to inclusion in the study. In 30 (85.7%) patients, the attending physician had communicated that their symptoms were of non-cardiac origin. Additionally, 18 (51.43%) patients were referred to a psychiatrist for symptom evaluation.

### 3.3. Interventional Diagnostic Procedure

Mean FFR measured on the IDP was 0.9 (0.88; 0.92), CFR was 2.1 (1.8; 3.1), and IMR 27 (18; 39) (Table 3). No adverse reactions were observed during the adenosine test.

Final diagnosis of MVA was confirmed in 19 (54.3%), VSA in nine (25.7%), microvascular angina in three (8.6%), and combination of MVA and VSA in three (8.6%) patients. Four (11.42%) patients returned normal results of IDP.

Acetylcholine provocation test was positive in 13 (37.1%) patients, of whom four developed microvascular spasm (three included in MVA group and one in combined endotype group), five (55.6%) experienced diffuse epicardial spasm, and four (33.3%) had a focal spasm.

During the acetylcholine provocation test, altogether 12 (34.28%) patients experienced transient bradycardia and two (5.7%) had a transitory second-degree atrioventricular block.

### 3.4. Medical Treatment Changes After the Intracoronary Physiology Testing

In patients with MVA, a significant increase in prescription rates of CCBs (26.3% to 94.7%, *p* < 0.001), antianginal treatment: ranolazine or trimetazidine (15.8% to 94.7%; *p* < 0.001), and BBs (42.1% to 73.7%; *p* = 0.05) was observed (Table 4). The VSA patient group also experienced increased prescription rates of CCBs (44.4% to 100%, *p* = 0.03), but the prescription rates of BBs decreased (88.8% to 22.2%, *p* = 0.015) and use of long-acting nitrates increased (0% to 77.8%, *p* = 0.002). There were no changes in prescribed treatment of the mixed INOCA endotype group and patients with normal IDP testing.

### 3.5. The Follow-Up and Quality of Life Assessment

Data from the three-month follow-up were available for all patients (Table 5). At the three-month follow-up, a significant improvement in SAQ-7 summary score was observed in the MVA and VSA groups (*p* < 0.001 and *p* = 0.007, respectively). Furthermore, both groups experienced improvements in all subsections of the SAQ-7 questionnaire (physical limitation, angina frequency, and QoL), with the MVA group reaching the most substantial improvements in the QoL score (40 to 100, *p* < 0.001). In contrast, SAQ-7 summary score and subsection values did not change in the mixed endotype group and patients with normal IDP results.

## 4. Discussion

The major findings in the current study are as follows: INOCA patients presented with a wide range of symptoms and decreased quality of life, which had resulted not only in recurrent doctor visits but also prolonged diagnostic process and extensive testing. Adjustment of medical therapy according to the exact INOCA endotype defined by invasive coronary physiology testing resulted in major improvements in physical limitations, angina frequency, and reported QoL in the MVA and VSA groups.

INOCA typically presents with a wide range of clinical symptoms, many of which are nonspecific. According to the results of several studies and a systematic review by Bairey-Merz et al., the INOCA patients typically report more symptoms compared with those with obstructive CAD [13,18,19,20,21]. Consistent with previous literature [16,21], 80% of our study population were women, reflecting the well-recognized observation that women with cardiac disease may present with non-specific or atypical symptoms compared to men [6]. Gulati et al. in cooperation with the INOCA International Group have reported the prevalence of chest pain in 92.9% of cases, dyspnea in 80.5%, and neurologic symptoms like confusion and dizziness in 77.4% in INOCA patients [16,22]. Our study found similar rates of these complaints, with a few notable exceptions; namely, we report very high rates of mental disorders. For example, a study by Schalkwijk et al. reported a 21% and 29% prevalence of at least moderate depression and anxiety, respectively [23]. In contrast, depression and anxiety rates in our study group were 34.3%. These findings are in line with the high prevalence (~6%) of clinical depression and even higher rates of untreated subclinical conditions like depressive cognitions and negative automatic thoughts (30%) in Latvia [24]. Depression, as a psychological stressor, has been implicated as both a cause and consequence of CMD [25]. Furthermore, we have noted that our patients during the invasive testing are more anxious, stressed, and emotional, which could suggest that depression could act as a trigger for CMD or vice versa. Furthermore, the long time to diagnosis likely contributes to the mood disorders, leading to a self-perpetuating cycle of worsening cardiac symptoms. Importantly, depression and anxiety are independently associated with reduced QoL [26], underscoring the need to treat these psychiatric conditions as distinct comorbidities rather than merely as accompanying symptoms. Effective management of INOCA in these cases should therefore involve a multidisciplinary approach that includes not only cardiac care but also psychosocial support. This may encompass psychotherapy, medical treatment for mood disorders, and coordinated teamwork among cardiologists, psychiatrists, psychologists, and other healthcare professionals. Addressing mental health comprehensively alongside cardiac care remains essential to improve overall patient outcomes and QoL.

In our study the average time of living with INOCA symptoms was 1–3 years and most diagnostic tests were negative or explained as false positives. This could be an additional reason why more than half of the patients in our study were told that their symptoms were not cardiac and referred to a psychiatrist.

Recent ESC Guidelines on chronic coronary syndrome and the COVADIS Group have provided detailed recommendations on the diagnostics and treatment of INOCA patients [7,8]. Nevertheless, implementation of these recommendations fully still faces many challenges, as the invasive physiology testing is relatively time-consuming and requires a tertiary level care interventional laboratory [27,28]. In our study, we applied the COVADIS diagnostic framework, which proved to be a practical and effective tool for identifying relevant patient subgroups.

The COVADIS criteria define the initial diagnostic requirement for INOCA as the presence of objectively confirmed ischemia detected through non-invasive imaging tests [7,8]. For some time, exercise ECG has been underrated for its relatively high false positive rates in obstructive CAD patients [29], but recent studies have been promising, showing up to 100% specificity for diagnosing CMD [30,31]. In contrast, patients with a vasospastic cause of chest pain frequently have normal stress test reports [27,32,33]. In our study the exercise ECG data were available for all patients. They were positive in all but five patients, who, however, had previously documented ischemic changes on a resting ECG during chest pain and were ultimately not diagnosed with MVA.

Invasive evaluation of INOCA is fundamental for tailoring treatment and achieving better quality of life outcomes [6,27]. In the cardiac catheterization laboratory, coronary vascular function can be evaluated ad hoc during the patient’s initial ICA. However, in our study all patients were referred for additional testing only after repeated out-patient visits and previously performed coronary angiograms, which also takes a longer time to reach diagnosis and successful treatment. Novel imaging techniques, such as optical coherence tomography, which allow plaque morphology assessment and offer mechanistic insights into the vessel adventitia, are currently under investigation and will hopefully expand our understanding of the underlying pathophysiology of INOCA [34,35]. All adverse events during IDP, such as bradycardia or atrioventricular block after acetylcholine infusion, were transient and did not require therapy adjustment. Consistent with previous findings [36], our results indicate that these reactions represent physiological responses to intracoronary acetylcholine administration rather than complications, confirming the excellent safety profile of acetylcholine provocation testing.

According to our study data, among patients with INOCA, MVA was the most common underlying cause of chest pain, followed by VSA in 25% of cases. The patient distribution in our study correlates with the largest randomized controlled trial—“CorMicA trial” (Stratified Medical Therapy Using Invasive Coronary Function Testing in Angina)—and its diagnostics of INOCA patients [14]. In contrast, an observational study in Portugal by Ferreira et. al. reported only 25% prevalence of MVA, while VSA was the most common INOCA endotype [37]. Additionally, authors found negative IDP results in 25% of the examined patients, while in our cohort these rates were significantly lower—11.42%—indicating a high prevalence of INOCA or stricter patient selection for IDP.

Optimal medical treatment plays a crucial role in reducing the burden of untreated and symptomatic patients. The new ESC Guidelines recommend medical therapy based on the underlying cause of INOCA [8]. This approach is largely supported by findings from the CorMicA trial, which demonstrated that identifying the specific INOCA endotype allows tailored treatment and leads to significant reductions in angina burden and improved QoL [14]. Our results are consistent with these observations, confirming that targeted, mechanism-specific therapy improves patient-reported outcomes in both MVA and VSA groups. The feasibility and safety of invasive diagnostic procedures in our study further support the routine clinical application of this strategy. Our results also complement earlier evidence from the WISE study [12], which first demonstrated that patients—particularly women—with angina and no obstructive coronary artery disease often experience persistent symptoms and reduced QoL despite the absence of flow-limiting lesions [12,19]. These findings emphasized the need for accurate diagnosis and individualized management aimed not only at risk assessment but also at improving daily functioning and alleviating angina symptoms, goals that align closely with the focus and outcomes of our study.

First-line therapy for patients with MVA includes beta blockers unless microvascular spasm is present, in which case these agents may worsen symptoms [38]. Current ESC recommendations emphasize a comprehensive anti-anginal approach that includes antianginal treatment such as ranolazine or trimetazidine, calcium channel blockers, and beta blockers in patients with MVA [8]. In contrast, beta blockers are contraindicated in vasospastic angina, where calcium channel blockers and nitrates remain the mainstay of therapy. More recently, the WARRIOR trial did not demonstrate significant differences in major adverse cardiovascular events or QoL between intensive medical therapy—focused mainly on ACE inhibitors and statins—and usual care, possibly reflecting the limited pharmacologic strategy applied [39]. In contrast, a recent Polish prospective study demonstrated marked improvements in QoL and angina severity after 12 months of individualized treatment in patients with INOCA [40], which aligns with our findings as well as those reported in CorMicA [14]. The ongoing iCorMicA trial currently has patient recruitment in progress, aiming to expand on these observations in a larger multicenter cohort [41].

Our findings suggest by that tailoring therapy to the underlying pathophysiology, significant improvements in QoL can be achieved even over a short follow-up. However, consistent with prior studies, these benefits have not yet translated into lower rates of major cardiovascular events, highlighting the need for further large-scale randomized trials to refine management strategies and improve long-term outcomes in this complex population.

### 4.1. Clinical Implications

Our results demonstrate major improvements in QoL after adjusting medical therapy according to IDP-tested endotype in MVA and VSA. These findings emphasize the need of routine implementation of IDP in selected patients with a high degree of suspicion for INOCA. Additionally, the lack of improvement in the patient group with the MVA and VSA mixed endotype highlights a research gap for further studies.

### 4.2. Limitations

The main limitation of the current study is its small sample size. Furthermore, its single-center design restricts generalizability, and future multicenter validation is important to confirm these findings across broader patient populations. Nevertheless, our data reflect the current national landscape, as our institution remains the sole center in the country performing comprehensive invasive testing for INOCA. The short follow-up period (3 months) was chosen to emphasize the early impact of tailored therapy on quality of life and the current study does not evaluate long-term outcomes. Additionally, this study did not include a control group and no randomization was performed. A controlled study with longer follow-up at 12 and 36 months, including assessment of adverse cardiovascular events, is currently ongoing.

## 5. Conclusions

Intracoronary physiology testing in patients with suspected INOCA is feasible and safe in clinical practice. Accurate identification of distinct endotypes enabled targeted, mechanism-specific therapy that led to major improvements in quality of life in patients with isolated MVA or VSA. The clear clinical benefits highlight the need for IDP implementation in the routine care of patients with cardiac chest pain when obstructive coronary artery disease is ruled out.

## Figures and Tables

**Table 1:** Baseline characteristics stratified according to invasive measurements.

Parameter	Total (n = 35)	MVA (n = 19)	VSA (n = 9)	Combination of MVA & VSA (n = 3)	Non-Cardiac (n = 4)	*p*-Value
Age (years)	61.00 (54.00; 69.00)	66.00 (57.00; 73.00)	54 (50.50; 60.00)	62.00 (58–66)	47.50 (45–73)	0.048
Sex (women)	28 (80.0%)	17 (89.5%)	6 (66.7%)	3 (100)	2 (50.0%)	0.16
Body mass index (kg/m^2^)	27.44 (23.89; 30.67)	27.99 (25.40; 30.86)	26.06 (23.10; 31.36)	26.03 (23.53–29.40)	22.34 (21.61–29.72)	0.20
**Comorbidities (n, %)**						
Arterial hypertension	30 (85.7%)	17 (89.5%)	8 (88.9%)	2 (66.7%)	3 (75%)	0.67
Atrial fibrillation	2 (5.7%)	0	1 (11.1%)	1 (33.3%)	0	0.08
Chronic heart failure	1 (2.9%)	0	1 (11.1%)	0	0	0.46
Dyslipidemia	35 (100%)	19 (100%)	9 (100%)	3 (100%)	4 (100%)	NS
Diabetes Mellitus (Type 1 and 2)	6 (17.1%)	2 (10.5%)	2 (22.2%)	1 (33.3%)	1 (25%)	0.69
Thyroid disorders	3 (8.6%)	1 (5.3%)	0	0	2 (50%)	0.090
Raynaud’s phenomenon	2 (5.7%)	1 (5.3%)	1 (11.1%)	0	0	NS
Autoimmune disorder	3 (8.6%)	2 (10.5%)	0	0	1 (25%)	0.62
Migraines	4 (11.4%)	2 (10.5%)	1 (11.1%)	0	1 (25%)	0.83
Depression/anxiety	12 (34.3%)	6 (31.6%)	3 (33.3%)	2 (66.7%)	1 (25%)	0.66
Smoking or ex-smoker	5 (14.3%)	0	4 (44.4%) ^†^	0	1 (25%)	0.014
**Laboratory data**						
Total cholesterol (mmol/L)	4.06 (3.25; 5.03)	4.40 (3.73; 5.17)	3.61 (3.03; 5.16)	3.74 (2.52–4.58)	3.64 (2.94–4.42)	0.3
Low-density lipoprotein cholesterol (mmol/L)	1.92 (1.49; 3.11)	2.14 (1.50; 3.53)	1.92 (1.69; 3.16)	1.37 (0.91–2.60)	1.54 (1.31–2.39)	0.29
High-density lipoprotein cholesterol (mmol/L)	1.53 (1.32; 1.8)	1.59 (1.44; 1.81)	1.28 (1.00; 1.52)	1.81 (1.11–2.13)	1.48 (1.34–2.03)	0.07
Non-high-density lipoprotein cholesterol (mmol/L)	2.45 (1.81; 3.42)	2.59 (2.13; 3.83)	2.09 (1.91; 3.69)	1.61 (1.41–1.61)	1.96 (1.52–2.89)	0.22
Triglycerides (mmol/L)	1.18 (1.02; 1.55)	1.23 (1.03; 1.67)	1.02 (0.9; 1.21)	2.39 (0.7–2.39)	1.29 (0.84–2.04)	0.31
Glucose (mmol/L)	5.5 (5.2; 5.7)	5.5 (5.2; 5.7)	5.5 (5.25; 6.15)	5.6 (4.5–7.2)	5.55 (4.6–6.7)	0.99
Glycated hemoglobin (%)	5.8 (5.5; 6.0)	5.8 (5.5; 5.9)	5.8 (5.75; 6.15)	5.5 (5.5–6.7)	5.55 (5.3–6.2)	0.55
Hemoglobin (g/L)	135 (129; 142)	136 (129; 143)	133 (124.5; 145)	132 (128–132)	134 (131–147)	0.89
Creatinine (µmol/L)	74 (66; 83)	73 (66; 82)	77 (69.5; 87)	76 (60–86)	70 (46–94)	0.74
Uric acid (mmol/L)	311 (236; 359)	340 (241; 362)	285 (231; 368)	278 (214–300)	234 (234–394)	0.60
Alanine transaminase (U/L)	28 (21; 37)	25 (19; 36)	32 (25; 50)	28 (21–28)	42 (24–66)	0.26
Thyroid-stimulating hormone (mU/L)	1.36 (0.81; 1.94)	1.756 (0.876; 2.180)	0.785 (0.53; 1.91)	1.322 (1.31–1.69)	1.130 (0.82–2.42)	0.22
Free thyroxine (µg/dL)	1.23 (1.1; 1.56)	1.23 (1.03; 1.56)	1.34 (1.09; 1.59)	1.37 (1.15–1.57)	1.14 (1.09–1.46)	0.79
Brain natriuretic peptide (pg/mL)	21.7 (15.36; 42.7)	24.12 (16.16; 46.25)	27.23 (15.12; 69.62)	37.56 (8.85–68.7)	15.24 (8.9–34.6)	0.52
**Symptoms (n, %)**						
Chest pain/discomfort/pressure	32 (91.4%)	17 (89.5%)	9 (100%)	3 (100%)	3 (75%)	0.62
Rest angina	11 (31.4%)	2 (10.5%)	8 (88.9%) ^†^	1 (33.3%)	0	<0.001
Shortness of breath	28 (80%)	16 (84.2%)	7 (77.8%)	3 (100%)	2 (50%)	0.39
Tachycardia/palpitations	8 (22.9%)	4 (21.1%)	1 (11.1%)	2 (66.7%)	1 (25%)	0.26
Fatigue/exhaustion	23 (65.7%)	14 (73.7%)	5 (55.6%)	3 (100%)	1 (25%)	0.16
Excessive sweating	10 (28.6%)	6 (31.6%)	3 (33.3%)	1 (33.3%)	0	0.74
Headaches	14 (40.0%)	9 (47.4%)	3 (33.3%)	2 (66.7%)	0	0.27
Sleep–wake disorders	4 (11.4%)	2 (10.5%)	1 (11.1%)	1 (33.3%)	0	0.60
Depression and anxiety	9 (25.7%)	5 (26.3%)	3 (33.3%)	1 (33.3%)	0	0.76

Data are presented as median (interquartile range) if n ≥ 5, median (minimum–maximum) if n < 5, and n (%). ^†^ Bonferroni post hoc analysis *p* < 0.05 vs. MVS. MVA: microvascular angina, VSA: vasospastic angina.

**Table 2:** Referral patterns and previous investigation before invasive physiology testing.

Years with INOCA Symptoms (n, %)	n = 35
<1 year	9 (25.7%)
1–3 years	19 (54.3%)
years	3 (8.6%)
>5 years	4 (11.4%)
**Clinical Assessment of Symptoms (n, %)**	
Explained that symptoms were not cardiac	30 (85.7%)
Referred to a psychiatrist for symptoms	18 (51.4%)
**Total visits of cardiologist due to symptoms (n, %)**	
1	5 (14.3%)
2–3	23 (65.7%)
>3	8 (22.9%)
**Total visits of emergency department (n, %)**	
1	12 (34.3%)
2–3	4 (11.4%)
>3	1 (2.9%)
**Age at INOCA diagnosis (years) (n, %)**	
<40	0 (0%)
41–50	5 (16.1%)
51–60	8 (25.8%)
61–70	11 (35.4%)
>70	7 (22.6%)
**Previous investigation tools (n, %)**	
ECG	35 (100%)
Ischemic ECG during the last year	5 (14.3%)
Exercise stress test	35 (100%)
Echocardiography	35 (100%)
Stress echocardiography	3 (8.8%)
CCTA	12 (34.3%)
Cardiac magnetic resonance imaging	5 (14.3%)
Cardiac SPECT or PET	4 (11.4%)
Invasive coronary angiography	30 (85.7%)
≥2 ICA for evaluation of symptoms	17 (48.6%)

Data are presented as n (%). ECG: electrocardiogram, CCTA: cardiac computed tomography angiography, SPECT: single-photon emission computed tomography, PET: positron emission tomography, ICA: invasive coronary angiography

**Table 3:** Data from intracoronary physiology testing.

Parameter	Total (n = 35)	MVA (n = 19)	VSA (n = 9)	Combination of MVA & VSA (n = 3)	Non-Cardiac (n = 4)	*p*-Value
**Coronary angiography**						
No lesions	30 (85.6%)	18 (94.7%)	7 (77.8%)	2 (66.7%)	3 (75%)	NS
Stenosis 20–50%	5 (14.3%)	1 (5.3%)	2 (22.2%)	1 (33.3%)	1 (25%)	NS
**Microcirculation measures**						
FFR	0.90 (0.88; 0.92)	0.90 (0.88; 0.92)	0.89 (0.84; 0.93)	0.90 (0.90–0.90)	0.90 (0.83–0.92)	0.66
CFR	2.1 (1.8; 3.1)	1.90 (1.70; 2.60)	3.10 (2.85; 3.75) ^†^	2.00 (0.9–2.00) *	2.65 (2.00–3.80)	0.003
CFR ≤ 2.5	19 (54.3%)	14 (73.7%)	0 ^†^	3 (100%) *	2 (50%)	<0.001
IMR	27 (18; 39)	38 (27; 40)	20 (16; 24) ^†^	38 (19–38)	23.50 (15.00–34.00)	0.025
IMR ≥ 25	20 (57.1%)	15 (78.9%)	1 (11.1%) ^†^	2 (66.7%)	2 (50%)	0.009
**Acetylcholine provocation test**						
Acetylcholine maximal dose (μg)	100	100	100	100		NS
Diffuse epicardial spasm	7 (20%)	0	5 (55.6%) ^†^	2 (66.7%)	0	<0.001
Focal epicardial spasm	4 (11.4%)	0	4 (100%) ^†^	0	0	0.009
Microvascular spasm	4 (11.4%)	3 (15.8%)	0	1 (33.3%)	0	0.36
Transient bradycardia	12 (34.29%)	8 (42.1%)	3 (33.3%)	0	1 (25%)	0.70
Second-degree atrioventricular block	2 (5.71%)	0	0	0	2 (10.5%)	NS

Data are presented as median (interquartile range) if n ≥ 5, median (minimum–maximum) if n < 5, and n (%). * Bonferroni post hoc analysis *p* < 0.05 vs. VSS. ^†^ Bonferroni post hoc analysis *p* < 0.05 vs. MVS. CFR: coronary flow reserve, FFR: fractional flow reserve, IMR: index of microvascular resistance, MVA: microvascular angina, NS: not significant, VSA: vasospastic angina

**Table 4:** Prescribed medical treatment before and after intracoronary physiology testing.

Parameter (n, %)	Total Baseline (n = 35)	Total Follow-Up (n = 35)	*p*-Value	MVA Baseline (n = 19)	MVA Follow-Up (n = 19)	*p*-Value	VSA Baseline (n = 9)	VSA Follow-Up (n = 9)	*p*-Value	Combined MVA & VSA Baseline (n = 3)	Combined MVA & VSA Follow-up (n = 3)	*p*-Value	Non- Cardiac Baseline (n = 4)	Non- Cardiac Follow-Up (n = 4)	*p*- Value
Aspirin	9 (25.7%)	9 (25.7%)	NS	5 (26.3%)	4 (21.2%)	NS	3 (33.3%)	3 (33.3%)	NS	1 (33.3%)	1 (33.3%)	NS	0	1 (25%)	NS
ACEI	15 (42.9%)	17 (48.6%)	0.63	8 (42.1%)	11 (57.9%)	0.52	3 (33.3%)	2 (22.2%)	NS	1 (33.3%)	1 (33.3%)	NS	3 (75%)	3 (75%)	NS
ARB	6 (17.1%)	4 (11.4%)	0.5	3 (15.8%)	3 (15.8%)	NS	2 (22.2%)	1 (11.1%)	NS	1 (33.3%)	0	NS	0	0	-
BB	18 (51.4%)	18 (51.4%)	NS	8 (42.1%)	14 (73.7%)	0.099	8 (88.8%)	2 (22.2%)	0.015	1 (33.3%)	1 (33.3%)	NS	1 (25%)	1 (25%)	NS
CCB	13 (37.1%)	32 (91.4%)	<0.001	5 (26.3%)	18 (94.7%)	<0.001	4 (44.4%)	9 (100%)	0.029	1 (33.3%)	3 (100%)	0.4	3 (75%)	2 (50%)	NS
Indapamide	4 (11.4%)	4 (11.4%)	NS	2 (10.5%)	3 (15.8%)	NS	1 (11.1%)	0	NS	1 (33.3%)	1 (33.3%)	NS	4 (100%)	4 (100%)	-
LANs	0	9 (25.7%)	0.004	0	2 (10.5%)	0.49	0	7 (77.8%)	0.002	0	0	-	0	0	-
AAT	3 (8.6%)	22 (6.9%)	<0.001	3 (15.8%)	18 (94.7%)	<0.001	9 (100%)	9 (100%)	-	0	3 (100%)	0.1	0	1 (25%)	NS
Statins	22 (62.9%)	35 (100%)	<0.001	13 (68.4%)	19 (100%)	0.02	5 (55.6%)	9 (100%)	0.08	2 (66.7%)	3 (100%)	NS	2 (50%)	4 (100%)	0.43

Data are presented as n (%). ACEI: angiotensin-converting enzyme inhibitors, AAT: anti-anginal treatment: ranolazine/trimetazidine, ARB: angiotensin receptor blocker, BB: beta blocker, CCB: calcium channel blocker, LANs: long-acting nitrates, MVA: microvascular angina, NS: not significant, VSA: vasospastic angina.

**Table 5:** The Seattle Angina Questionnaire (SAQ-7) results before and 3 months after the testing.

Parameter (n, %)	Total Baseline (n = 35)	Total Follow-Up (n = 35)	*p*-Value	MVA Baseline (n = 19)	MVA Follow-Up (n = 19)	*p*-Value	VSA Baseline (n = 9)	VSA Follow-Up (n = 9)	*p*-Value	Combined MVA & VSA Baseline (n = 3)	Combined MVA & VSA Follow-Up (n = 3)	*p*-Value	Non- Cardiac Baseline (n = 4)	Non- Cardiac Follow-Up (n = 4)	*p*- Value
Total SAQ	71.43 (68.57; 71.43)	82.86 (80; 82.86)	<0.001	50 (50; 60)	71.41 (68.57; 74.29)	<0.001	50 (50; 60)	71.43 (68.57; 74.29	0.007	68.57 (60–71.43)	80 (77.14–82.86)	0.11	64.29 (60–71.43)	78.57 (74.29–80)	0.11
SAQ physical limitation	86.67 (80; 86.67)	93.33 (86.67; 93.33)	<0.001	86.67 (86.67; 86.67)	93.33 (86.67; 100)	<0.001	86.67 (80; 86.67)	93.33 (86.67; 100)	0.016	86.67 (66.67–86.67)	86.67 (86.67–93.33)	0.32	76.67 (60–86.67)	86.67 (86.67–93.33)	0.066
SAQ angina frequency	80 (80; 80)	100 (90; 100)	<0.001	80 (80; 90)	100 (90; 100)	0.001	80 (80; 90)	100 (90; 100)	0.014	80 (80–80)	100 (90–100)	0.102	80 (70–90)	90 (80–90)	0.083
SAQ QoL	40 (30; 40)	50 (50; 50)	<0.001	40 (30; 40)	100 (90; 100)	<0.001	40 (35; 40)	60 (50; 60)	0.006	30 (30–40)	50 (50–50)	0.102	40 (20–40)	50 (50–60)	0.066

Data are presented as median (interquartile range) if n ≥ 5, median (minimum–maximum) if n < 5, and n (%). SAQ—The Seattle Angina Questionnaire, QoL—quality of life, MVA: microvascular angina, VSA: vasospastic angina.

## Data Availability

The data presented in this study are available upon request from the corresponding author.

## Abbreviations

The following abbreviations are used in this manuscript:

ACEI	angiotensin-converting enzyme inhibitor
ARB	angiotensin receptor blocker
BB	beta blocker
CAD	coronary artery disease
CCB	calcium channel blocker
CCTA	cardiac computed tomography angiography
CFR	coronary flow reserve
CMD	coronary microvascular dysfunction
COVADIS	Coronary Vasomotor Disorders International Study
ECG	electrocardiogram
ESC	European Society of Cardiology
FFR	fractional flow reserve
ICA	invasive coronary angiography
IDP	interventional diagnostic procedure
IMR	index of microvascular resistance
INOCA	ischemia with non-obstructive coronary arteries
MVA	microvascular angina
QoL	quality of life
SAQ-7	Seattle Angina Questionnaire 7
VSA	vasospastic angina
